# Sharp Skills or Snipping Struggles? Qualitative Paper-Cutting Performance in 5- to 10-Year-Old Children Using Hands-On!

**DOI:** 10.3390/bs15040489

**Published:** 2025-04-08

**Authors:** Leila Faber, Esther Hartman, Suzanne Houwen, Marina M. Schoemaker

**Affiliations:** 1 Department of Human Movement Sciences, University Medical Center Groningen, University of Groningen, 9713 AV Groningen, The Netherlands; e.hartman@umcg.nl (E.H.); m.m.schoemaker@umcg.nl (M.M.S.); 2 Inclusive and Special Needs Education Unit, Faculty of Behavioral and Social Sciences, University of Groningen, 9712 TS Groningen, The Netherlands; s.houwen@rug.nl

**Keywords:** motor skill development, fine motor skills, paper cutting, scissor use, typical development, observational, Hands-On!

## Abstract

This study examined age-related changes in qualitative paper-cutting performance of typically developing children aged 5 to 10 years. Using the Hands-On! observation tool, we analysed 178 (85 boys; Mage 8.06 years, SD ± 1.58) children’s performance on the DCDDaily paper cutting task. Paper cutting involves several intra-task components, such as grip type and cutting movements, each comprising multiple observable actions that reflect a child’s qualitative performance (e.g., small or large cutting movements). We assessed the differences in the occurrence of these actions within each intra-task component across age groups, along with task duration and mistakes. Our findings revealed significant age-related differences in the qualitative performance of multiple actions within the intra-task components. Three distinct developmental patterns emerged: progression, variability in progression, and stability. Notably, these qualitative differences were observed even when quantitative measures, such as duration and mistakes, showed ceiling effects, highlighting the ability of qualitative assessments to capture nuanced developmental changes. This study provides valuable insights into the development of paper-cutting skills, emphasising the importance of incorporating qualitative analysis into motor skill assessments. Future research should explore the qualitative performance of children with both typical and atypical motor development to further understand the complex interplay of factors influencing fine motor skill performance.

## 1. Introduction

Beginning in early childhood, children develop fine motor skills that are used to perform everyday tasks. Fine motor skills serve evolving purposes across various life stages: infants use them to explore, toddlers refine them for more precise object manipulation and (bi-manual) coordination, and school-aged children further develop their fine motor skills for self-care and the ability to use tools such as pencils and scissors, fostering greater independence and autonomy (Gabbard, 2021; Haywood & Getchell, 2014; A. Henderson & Pehoski, 2005; Sorgente et al., 2021). Fine motor skills can be described as activities of the hands and fingers that involve the coordination of small movements (Cuffaro, 2011).

Paper cutting is a common fine motor skill that most children perform at home or school and continue to use throughout their lives, making it highly relevant to everyday activities (Ratcliffe et al., 2011). Furthermore, paper cutting incorporates elements also used in other fine motor skill tasks, and, in other words, is transferable to tasks like handwriting, paper folding, buttoning clothes, and preparing food (Rodger et al., 2003). This transfer refers to how skills learned in paper cutting can influence the acquisition of similar skills, particularly in terms of hand strength, coordination, and, in most tasks, bi-manual hand use (Davids et al., 2008; Rodger et al., 2003). As a skill learned early in childhood, it remains in use throughout life, making it valuable for studying fine motor development. Finally, paper cutting is often an important goal in occupational therapy, especially for children with Developmental Coordination Disorder (DCD) (Zwicker et al., 2012). The development of fine motor skills, including paper cutting, is neither linear nor uniform, even in typically developing children (Adolph et al., 2008). Newell’s constraints theory (Newell, 1986) provides a useful framework for understanding the factors influencing this variability by highlighting three types of constraints: task, environmental, and individual constraints. First, task constraints, such as specific requirements to cut along a line, adhere to time limits, or achieve a particular level of accuracy, can influence how a child approaches and performs the activity. For example, the child is asked to prioritise precision over speed, demonstrating a slower yet more accurate cutting style, or vice versa, illustrating the speed-accuracy trade-off, which is a result of a change in individual constraint based on the given task constraint (Latash, 2012). Second, environmental constraints, such as the availability of crafting materials, the physical setup of the testing area, the presence of peers, and social and cultural norms, can affect a child’s behaviour during a task. Distractions or supportive contexts may alter performance, emphasising how situational factors at the time of measurement influence observed behaviour (Adolph & Hoch, 2019; Haywood & Getchell, 2014). Finally, individual constraints, including factors such as age, prior experience, and intrinsic motivation, introduce variability both within the same child across measurements and between different children. For instance, a child’s willingness to engage in a task or their emotional state on a given day can impact their performance (Adolph & Hoch, 2019; Haywood & Getchell, 2014). Building on Newell’s constraints theory, the Dynamical Systems Theory (DST; Thelen & Smith, 2006) highlights the degrees of freedom in a child’s arms and hands, emphasising the diversity of movement strategies available to complete similar tasks. This diversity leads to variations in qualitative performance, even when quantitative outcomes, such as task duration, are identical. For instance, two children may complete a cutting task in the same time, yet one may exhibit smooth, controlled movements utilising a lot of degrees of freedom while the other displays inconsistent or jerky motions, freezing degrees of freedom (Bernstein, 1967; Guimarães et al., 2020). Observing these qualitative aspects offers valuable insights into motor skill development, revealing compensatory strategies or progress that might go unnoticed in quantitative assessments.

Paper cutting is a complex fine motor skill that requires a range of physical, cognitive, and neurological abilities (Edwards, 2018b; A. Henderson & Pehoski, 2006; Ratcliffe et al., 2011). Cutting paper with scissors depends on trunk and shoulder stability, wrist and hand muscle development, cognitive readiness, including motivation, self-regulation, sequencing, motor planning, and neurological maturity (e.g., integration of primitive reflexes for bilateral hand use) (Edwards, 2018b; A. Henderson & Pehoski, 2006; Ratcliffe et al., 2011). These abilities not only allow cutting to occur, but also shape the qualitative aspects of the performance. For example, the choice of grip type on the scissors reflects cognitive understanding of the requirements of the task, guiding precise tool movements (Rosenbaum et al., 2012). Effective cutting requires independent yet coordinated movements of both hands and accurate use of the thumb, index finger, and middle finger. Additionally, tactile and proprioceptive information are essential for force adjustment while cutting with one hand and guiding the paper with the other hand in this bi-manual task. During the early development of paper cutting, overflow (i.e., the unintentional movements that might occur alongside voluntary movements) can be visible as a result of the neurological connection between the hand and mouth, reflected as the opening and closing of the mouth simultaneously with the opening and closing of the scissors (Edwards, 2018b).

By exploring the specific actions or paper cutting, such as grip type, movement coordination, and bi-manual hand use, we can better understand the nuances of qualitative motor performance and its developmental progression. However, assessing fine motor skills like paper cutting often relies on quantitative measures, such as task duration or error counts (Van der Linde et al., 2013). While these measures are commonly used and provide standardised comparisons between children with typical and atypical (motor) development, they fail to capture the quality of performance (Hadwin et al., 2023). To address this gap, the Hands-On! observation tool was developed (Faber et al., 2024, 2025). Hands-On! is a qualitative observation tool for observing the fine motor skills of 5- to 10-year-old children, including paper folding, writing, and paper-cutting tasks. It consists of intra-task components, such as arm movements and grip types, which contribute to overall performance. Each intra-task component (e.g., cutting movements) consists of multiple possible actions that reflect the qualitative performance of a child (e.g., small or large cutting movements). Observing what actions are most prevalent within intra-task components in different age groups enables us to describe developmental patterns (Gabbard, 2021; Goodway et al., 2020; Haywood et al., 2012; Haywood & Getchell, 2014; Roberton et al., 2017). Understanding these developmental patterns provides a nuanced view of fine motor development, allowing researchers and practitioners to uncover specific aspects of performance that are indicative of motor difficulties, such as inefficient motor patterns or compensatory strategies.

Despite its importance in everyday tasks, paper cutting has been understudied in children, particularly in terms of qualitative performance. This study aims to address this gap by describing age-related changes in paper cutting among 5- to 10-year-old typically developing children using the Hands-On! observation tool. By thoroughly mapping the developmental trajectory of paper cutting by focusing on its qualitative aspects, this explorative research seeks to unravel developmental patterns that contribute to a deeper understanding of paper-cutting skills. This could provide a benchmark for comparing the performance of children with motor skill difficulties to typically developing children, ultimately informing diagnosis and more targeted interventions.

## 2. Materials and Methods

This study is part of the research initiative ‘Uniek in je Motoriek’ [Being unique with regard to your motor skills] from the Department of Human Movement Sciences at the University Medical Center Groningen (UMCG) and the University of Groningen in the Netherlands. Ethical approval for this study was obtained from the Ethics Review Committee of the UMCG (Research Registration number: 202100788). Following approval, primary schools were invited to participate in the research. Schools that agreed to participate distributed study invitations to parents, along with an information letter and video explaining the research. Participation was dependent on receiving a signed informed consent form from the parent, caregiver, or guardian.

The eligibility criteria required participants to be between 5 and 10 years of age and enrolled in mainstream Dutch primary schools. To ensure that the study included typically developing children, the parents completed a brief screening questionnaire. This was used to exclude children with physical disabilities, developmental disorders (e.g., intellectual disability, ADHD, Autism Spectrum Disorder, Developmental Coordination Disorder), or sensory impairments.

### 2.1. Participants

The study included 178 children (85 boys; 47.8%) aged 5 to 10 years, with a mean age of 8.06 years (SD ± 1.58), from three primary schools across the Netherlands. The descriptive statistics for age, sex, and handedness are presented in Table 1. Handedness was assessed by asking each child to draw a line on a piece of paper, with the pen placed in the centre of the paper, and observing which hand they used to complete the task.

### 2.2. Hands-On!

The Hands-On! (Faber et al., 2024, 2025) qualitative observation tool is a standardised instrument developed to assess the fine motor performance of children aged 5 to 10 years, see the Supplementary Material. Unlike tools that primarily focus on task outcomes, Hands-On! emphasises the qualitative aspects of how tasks are performed, offering detailed observations of various actions within multiple intra-task components across fine motor skills, such as paper folding, writing, and cutting. It was specifically designed for occupational and physical therapists to provide a unified and structured framework for evaluating the qualitative performance of children with fine motor challenges. Hands-On! provides both qualitative observations and a total proficiency score, distinguishing between lower and higher levels of qualitative performance. For the current study, Hands-On! was employed to analyse the qualitative observations of the paper-cutting performance (see Table 2 for a list of intra-task components and their actions).

### 2.3. DCDDaily Paper-Cutting Task

The DCDDaily is a valid and reliable instrument for assessing daily living skills in both children with DCD and typically developing children aged 5 to 8 years (Van der Linde, 2014; Van der Linde et al., 2013). It consists of 18 tasks divided into three domains: Self-care and Self-maintenance (11 tasks), Productivity and Schoolwork (5 tasks), and Leisure and Play (2 tasks) (Van der Linde, 2014).

**Figure 1:**
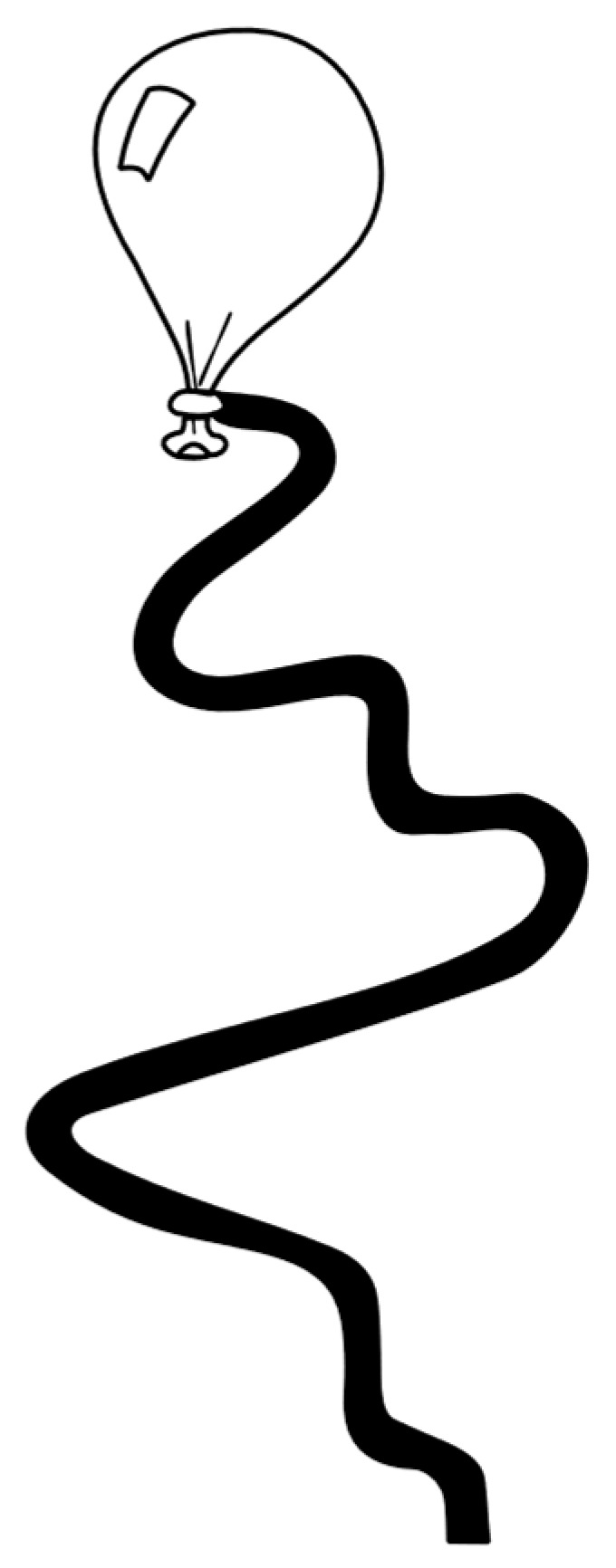
Cutting task of the DCDDaily. Figure from: DCDDaily Manual, obtainable via http://www.dcddaily.com/home/downloads/, accessed on 9 January 2025.

For this study, we focused on the paper-cutting task from the Productivity and Schoolwork domain, adhering to the instructions outlined in the DCDDaily manual, available at http://www.dcddaily.com, accessed on 9 January 2025 (Van der Linde, 2014). This task requires children to cut along a wavy line, assessing both the speed and accuracy of their performance (see Figure 1). The DCDDaily was used exclusively to ensure consistency among participants in performing the same paper-cutting task; however, its outcomes were not utilised in the current study.

### 2.4. Procedure

Each child was assessed individually in a quiet room at their school by a trained assessor, either a master’s student in human movement sciences or the primary researcher (LF). As part of the Uniek in je Motoriek project, additional fine motor tasks of the DCDDaily were also administered. The assessments were recorded on video for further analysis. Completing the entire assessment took approximately 15 to 20 min per child.

The video recordings of all 178 children performing the paper-cutting task were analysed by the primary researcher using the Hands-On! observation tool. The full duration of task performance from start to finish was recorded for each child. Observation and scoring of the paper-cutting task typically required around 2 min per video.

### 2.5. Data Analysis

The data were analysed using the Statistical Package for the Social Sciences (SPSS, Version 27, IBM, Armonk, NY, USA). The level of significance was set at *p* < 0.05. Prior to the analysis, normality tests were conducted, revealing that several variables were non-normally distributed. Consequently, non-parametric analyses were performed.

**Figure 2:**
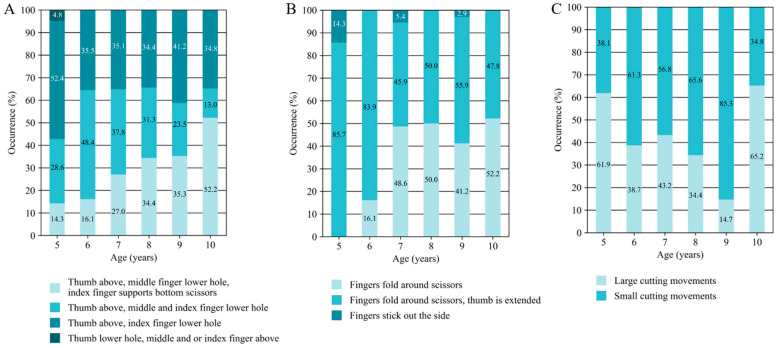

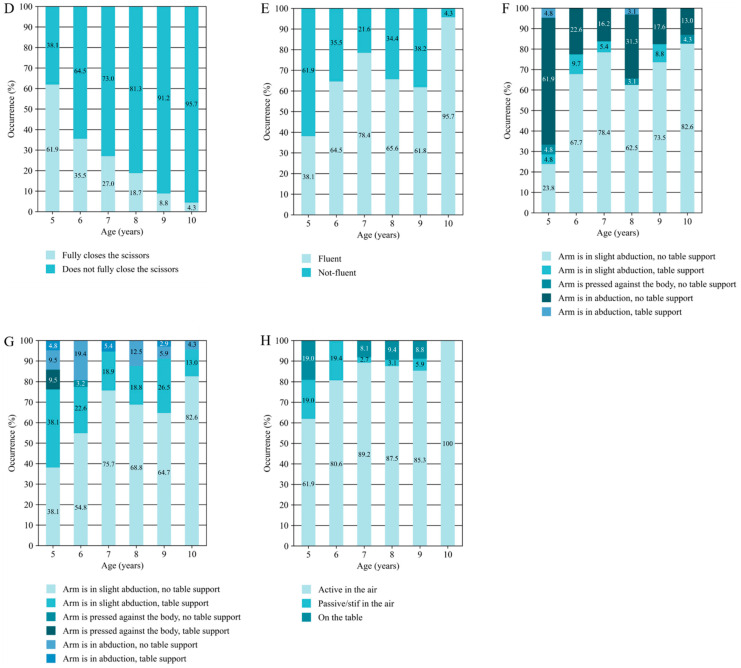
Percentages of the occurrence of actions within grip type scissors (**A**), grip type fingers (**B**), cutting movement (**C**), closing the scissors (**D**), fluency (**E**), arm position of the working hand (**F**), arm position of the supporting hand (**G**), and supporting hand (**H**) during the paper-cutting task.

To determine whether the occurrence (%) of the different actions differed per intra-task component across age, Kruskal–Wallis tests were performed for each action. For actions that showed significant differences in the Kruskal–Wallis tests, developmental patterns were explored by visual inspection of the percentages of occurrence for each action across the age groups, as presented in Figure 2. This descriptive approach provided additional insights into the nature of the observed differences, facilitating a more nuanced interpretation of the results.

Finally, to determine the differences between age groups in the quantitative aspects of the paper-cutting task (duration and number of mistakes), Kruskal–Wallis tests were performed. Post-hoc Mann–Whitney U tests with Bonferroni correction were used to explore the pairwise group differences.

## 3. Results

**Figure 3:**
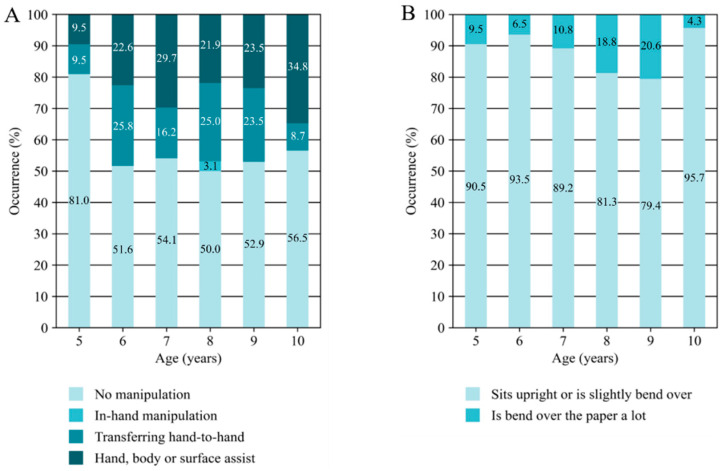
Percentages of the occurrence of actions within manipulation (**A**) and posture (**B**).

Table 3 presents the results of the Kruskal–Wallis analyses conducted across all age groups, highlighting the test statistics, significance levels, and effect sizes for intra-task components and their associated actions, focusing specifically on significant age-related differences. In the following paragraphs, the direction of the significant age group differences, as well as a visual overview of the directions (see Figure 2), are described. If only two actions are part of an intra-task component, both directions (opposite) are described as such. For the intra-task components, Posture and Manipulation, no significant differences were found (Figure 3). Each figure shows the percentage of occurrence of each action within an intra-task component across ages. Following this, the results of the analysis of the quantitative data (duration and number of mistakes) are presented.

### 3.1. Grip Type: Scissors

Within the component grip type on the scissors, the percentage of occurrence of the action ‘Thumb in the top hole, middle finger in the bottom hole, index finger supports’ was significantly different across the age groups, with the lowest prevalence in 5-year-olds (14.3%), which gradually increased across the age groups to a prevalence of 52.2% in 10-year-olds (Figure 2A).

### 3.2. Grip Type: Fingers

Within the component grip type fingers, the percentage of occurrence of the actions ‘Fingers fold around the scissors’, ‘Fingers fold around the scissors but thumb is extended’, and ‘Fingers stick out the side’ was significantly different across the age groups. Younger children (around 5 to 6 years old) more frequently exhibited actions, in which the thumb was extended, or all fingers were sticking out from the scissors. Notably, all the 5-year-olds displayed one of these two behaviours. In contrast, older children (around 7 years and older) more often demonstrated either thumb extension or all fingers folding securely around the scissors (Figure 2B).

### 3.3. Cutting Movement

Within the component cutting movement, the percentage of occurrence of both possible actions ‘Big cutting movements’ and ‘Small cutting movements’ was significantly different across the age groups. Here, we observed an increase in small cutting movements and a decrease in big cutting movements from age 5 to age 9-years-old. In 10-year-olds, we again observed an increase in big cutting movements (Figure 2C).

### 3.4. Closing the Scissors

Significant differences between age groups in the percentage of occurrence were found within the component closing the scissors for both actions (‘Closing the scissors completely’ and ‘Does not fully close the scissors’). Specifically, the occurrence of fully closing the scissors decreased with age, while the occurrence of not fully closing the scissors increased (Figure 2D).

### 3.5. Fluency

For component fluency, significant differences were found across age groups for both the actions ‘Fluent’ and ‘Not fluent’. In general, fluent cutting behaviour increased and nonfluent cutting decreased with age, although a slight decrease in fluent cutting was observed in the 8- and 9-year-old age groups (Figure 2E).

### 3.6. Arm Position Working Hand

Within the component arm position of the working hand, significant differences were observed across age groups in the occurrence of the actions ‘Slight abduction, no table support’ and ‘Arm in abduction, no table support’. Specifically, 5-year-olds displayed a lower frequency of ‘Slight abduction, no table support’ and a higher frequency of ‘Arm in abduction, no table support’ compared to all other age groups (Figure 2F).

### 3.7. Arm Position Supporting Hand

Within the component arm position of the supporting hand, significant differences were observed across age groups in the occurrence of the actions ‘Slight abduction, no table support’ and ‘Pressed against the body, supported on the table.’ The action ‘Arm in slight abduction, no table support’ became increasingly prevalent with age, although a slight decrease was noted in the 8- and 9-year-old groups. In contrast, the action ‘Arm pressed against the body, supported on the table’ was observed exclusively in 5-year-old participants (Figure 2G).

### 3.8. Supporting Hand

The percentages of occurrence of the actions ‘Active in the air’ and ‘Passive or stiff in the air’ of the supporting hand were significantly different across age groups. The ‘Active in the air’ action was observed in 61.9% of 5-year-olds, with its prevalence gradually increasing as children got older, reaching 100% among 10-year-olds. Conversely, the actions ‘Passive or stiff in the air’ of the supporting hand was more common in younger children, decreasing with age, although a slight increase was observed in the 8- and 9-year-old groups (Figure 2H).

### 3.9. Duration and Number of Mistakes

The Kruskal–Wallis test for task duration revealed a significant difference between the groups (H(5) = 14.059, *p* = 0.015). Post-hoc analysis (Mann–Whitney U tests) indicated the following significant differences: 5-year-olds differed significantly from 9-year-olds (U = 43.449, *p* = 0.036) and 10-year-olds (U = 54.404, *p* = 0.007), indicating that 5-year-olds were significantly slower compared to 9- and 10-year-olds. No other significant differences were observed between the age groups.

The Kruskal–Wallis test for the number of mistakes revealed a significant difference between groups (H(5) = 45.677, *p* < 0.001). Post-hoc analysis (Mann–Whitney U) showed the following significant differences: 5-year-olds made significantly more mistakes compared to children aged 7 (U = 43.943, *p* = 0.018), 8 (U = 65.369, *p* < 0.001), 9 (U = 65.413, *p* < 0.001), and 10 years (U = 83.010, *p* < 0.001). Similarly, 6-year-olds made more mistakes than children aged 8 (U = 39.460, *p* = 0.024), 9 (U = 39.504, *p* = 0.020), and 10 years (U = 57.101, *p* < 0.001). Finally, the 7-year-olds made significantly more mistakes than the 10-year-olds (U = 39.067, *p* = 0.045).

## 4. Discussion

This study contributes to the limited research on paper-cutting performance by exploring age-related changes in the qualitative aspects of paper cutting among typically developing children aged 5 to 10 years. Using the Hands-On! observation tool, we identified qualitative differences in how children approached paper-cutting tasks, offering insights into paper cutting beyond traditional measures of task duration or error counts. Our findings reveal clear developmental differences in several actions within the intra-task components of scissor use during paper cutting across age groups.

In general, we identified three patterns: (1) progression, (2) variability in progression, and (3) stability. The progression pattern is characterised by an age-related shift, where the most proficient actions become more frequent, while the occurrence of less proficient actions gradually decreases. In contrast, the variability in the progression pattern begins with a broader range of actions in younger children, particularly among 5-year-olds, who exhibit more than just the two prominent actions seen in the progression pattern. Over time, this variability decreases, leading to a similar increase in the most proficient actions and a decrease in less proficient actions as age increases. Finally, the stability pattern involves one or more actions, the occurrence of which remained relatively consistent across all age groups.

The most common pattern observed was progression. First, we observed an increase in the ‘Thumb in the top hole, middle finger in the bottom hole, and index finger supporting’ (Grip type scissors) with age, aligning with previous findings in younger children up to 6 years old (Case-smith & O’Brien, 2010; Edwards, 2018b; Ratcliffe et al., 2007). Similarly, in the grip type of the fingers (Grip type fingers), we observed an increase in the folding or flexing of the fingers to stabilise the scissors, while the loose grip type, where the fingers poke through the holes of the scissors, decreased (Case-smith & O’Brien, 2010; Edwards, 2018b; Mitchell et al., 2012). This results in more stability when holding the scissors with increasing age and could be one of the reasons fluency increased as well over the age groups.

In addition to the grip type, we observed an interplay between cutting movements and the closure of scissors, both of which exhibited a progression pattern. However, we did not formally test the relationship between these components; therefore, this observation should be interpreted with caution. Smaller cutting movements became more common with age, accompanied by a decrease in complete scissor closures. These adaptations may promote fluency by minimising overshooting and enabling a smoother and continuous cutting motion. Interestingly, in 10-year-olds, there was a preference for larger cutting movements coupled with not fully closing the scissors. This approach appears to be linked to the active support of the supporting hand, which stabilises and steers the paper. While small scissor movements aid in directional control, larger scissor movements optimise blade use, with the supporting hand guiding the paper along the curved lines, thereby promoting smoother cutting. Moreover, the increased use of active support from the non-dominant hand (Supporting hand) was accompanied by an increase in fluent movements.

The greatest variation within the age groups, particularly among 5-year-olds, was observed in the arm position (both in the working and supporting hands). In the youngest group, all possible actions occurred without a clear preference for one action, exhibiting variability in the progression pattern, with notable differences in working hand strategies. One action with a higher occurrence in 5-year-olds compared to older children was abduction of the working hand, often coupled with passive support of the supporting hand. This coupling can be explained by DST (Thelen & Smith, 2006), suggesting that reducing the degrees of freedom helps improve coordination (Bernstein, 1967; Guimarães et al., 2020). When the supporting hand provides only passive support, it effectively freezes the degrees of freedom. Consequently, navigating scissors around corners requires steering from a different joint. To maintain minimal degrees of freedom, movement appears to originate primarily from the shoulder, while the wrist and elbow remain relatively still, resulting in increased shoulder abduction to compensate for these movement restrictions. Another action we observed almost exclusively in our 5-year-old group was the adduction of both the working and supporting hands. This may again align with DST (Thelen & Smith, 2006), suggesting that reducing the degrees of freedom, in this case from the shoulder, helps improve coordination (Bernstein, 1967; Guimarães et al., 2020). As a result of freezing the movement from the shoulder, the steering motions of the scissors were probably performed with the wrists. In contrast to 5-year-olds, older children predominantly adopted a more neutral position, with the arm slightly abducted and without table support, and often with an active supporting hand which released degrees of freedom and allowed for greater flexibility (Bernstein, 1967).

The intra-task components Manipulation and Posture showed no significant differences across the age groups, revealing a stability pattern. The most common actions were ‘no manipulation’ (Manipulation) and ‘sitting upright or slightly bend over’ (Posture). Although ‘no manipulation’ was most prevalent in all age groups, other actions such as ‘hand, body, or surface assist’ and ‘transferring hand-to-hand’ were also visible in the intra-task component Manipulation and remained relatively stable in all age groups. Although in-hand manipulation generally requires more coordination than bi-manual manipulation, the absence of manipulation (no manipulation action) is more difficult to interpret. No manipulation could indicate that children initially grasped the scissors correctly, eliminating the need for adjustments, or that they failed to recognise the need to adjust an incorrect grip to enhance performance. Although an earlier study suggested a progression from bi-manual to in-hand manipulation in general (Edwards, 2018a), in practice, simpler and quicker strategies, such as repositioning the scissors with the use of the other hand (Hand, body, or surface assist), often achieve the goal more efficiently. For Posture, the consistency across all age groups may result from the formal assessment setting, as static and dynamic sitting postures begin to develop in infancy and are thus already well-trained in school-aged children (Gabbard, 2021). In a formal setting with an unfamiliar researcher, children may be more motivated to conform to perceived social norms and expectations, including adherence to proper posture (Baumeister & Leary, 2017). Additionally, children are accustomed to sitting for prolonged periods during regular classes at school (Geldhof et al., 2007), which may explain why they could maintain a proper posture during the brief assessment period.

Our research demonstrated the occurrence of various developmental patterns (progression, variability to progression, and stability), as no single predominant pattern was present in all intra-task components of paper cutting. DST posits that development unfolds as a non-linear process (Thelen & Smith, 2006), a concept supported by research highlighting diverse developmental patterns in motor development, including linear, accelerating, step-like, and variable progressions (Adolph et al., 2008). While our cross-sectional design cannot directly reveal developmental trajectories, the observed variety of patterns suggests that factors beyond age influence skill acquisition. This implies that the movement does not progress steadily or predictably. Instead, even a slight yet pivotal change within the individual, environment, or task can precipitate a systemic shift, leading to the emergence of new motor behaviours (Newell, 1986). For instance, the uniformity of materials suggests that the hand-to-material ratio may have affected the grip type; as hand size increases (Bear-Lehman et al., 2002), it may become impossible to fit both the index and middle fingers into the scissor holes simultaneously. What is intriguing is that even within a single task, we observed various developmental patterns between different age groups. This highlights the importance of considering individual differences in motor skill performance, even when performing the same task.

### 4.1. Discontinuation in Development

Although most results align with the expectation that older children show more proficient behaviour compared to younger children, or that stable behaviour is shown for all age groups, some unexpected findings emerged. Despite the general trend of an increase in more proficient actions and a decrease in less proficient actions, this progression did not always align with our initial expectations. In most of these components, we found a drop or stagnation in the progression in children aged 8- or 9-years old, and to a lesser extent in 7-year-olds. Earlier research has found similar discontinuation in the development of motor skills in children (Hay, 1979; Meulenbroek & van Galen, 1988; Meulenbroek & Van Galen, 1986). Hay (1979) studied open-loop reaching movements and found a shift from predominantly ballistic movements at age 5 to less fluent, visually guided movements by age 7 while reaching. Meulenbroek and Van Galen observed a discontinuity in the development of handwriting during primary school. Specifically, they found a decline in handwriting performance during the transition from grade two (8-year-olds) to grade three (9-year-olds), marked by slower writing speeds and increased dysfluency. This decline, however, reversed for children aged 10 to 12, who demonstrated improvements in handwriting fluency and speed (Meulenbroek & van Galen, 1988; Meulenbroek & Van Galen, 1986). These findings underscore variations in developmental scenarios, highlighting the occurrence of discontinuation in development across different motor skills in both previous research and the current study. Similarly, several actions within the intra-task components showed development that did not follow a linear development from least to most proficient but displayed fluctuations of actions within the intra-task components across ages. The temporary dysfluency of movements around 7- to 9-years of age is proposed to be related to the requirement for effective integration of feedback control (Hay, 1984). In the paper-cutting task of this study, feedback control may be important for understanding how cutting actions affect the task of cutting within the lines. The ability to regulate the closure of the scissors or adjust the paper to ensure that cutting remains within the lines, as defined by the task, may be key for task completion. In future research, it may be valuable to conduct specific research on feedback control in paper cutting.

### 4.2. Duration and Number of Mistakes

The quantitative results for both task duration and number of mistakes suggest the presence of ceiling effects in paper-cutting performance, albeit at slightly different ages. For task duration, a possible ceiling effect appears to emerge around age 6. While 5-year-olds were significantly slower than 9- and 10-year-olds, no significant difference was observed between 5- and 6-year-olds or among any groups older than 6. This pattern suggests that the most substantial gains in speed occur relatively early in development, likely before age 6. From age 6 onwards, children’s task duration plateaued, with no further statistically significant reductions in completion time. A similar, though slightly later, pattern was evident for the number of mistakes. A possible ceiling effect for this variable appeared to begin at around 8 years of age. Significant differences in the number of mistakes were found between younger children (5, 6, and 7-year-olds) and older children (8, 9, and 10-year-olds). However, no significant differences in mistake frequency were observed among the 8-, 9-, and 10-year-old groups. This suggests that by age 8, children achieved a level of proficiency where they made a similarly low number of mistakes, and after that age, the error rate did not significantly reduce.

First, ceiling effects on the DCDDaily cutting task for typically developing children are somewhat expected. This is primarily because the DCDDaily was specifically developed to assess difficulties in daily life activities for children with DCD, a group that typically performs less well than typically developing children (Van der Linde, 2014; Van der Linde et al., 2013). As with other tests, it is anticipated that children without motor skill difficulties would score at the upper end of the test. Additionally, the DCDDaily was designed for children aged 5 to 8 years, but our study included children aged 9 and 10 years (Van der Linde, 2014; Van der Linde et al., 2013). This may help explain the ceiling effect observed in the number of mistakes at age 8, as the task was originally designed for children up to this age. We chose to include 9- and 10-year-old children in our study because these age groups are also important for understanding motor skill development, especially given that children with DCD are often diagnosed within this age range. Moreover, we aimed to determine whether the qualitative development of paper cutting would also exhibit a ceiling effect or continue to evolve beyond the age of 8. By including older children, we could assess whether qualitative motor skill performance plateaus or whether further changes occur, providing a clearer picture of developmental trajectories. Since the primary focus of our study was the qualitative aspect of paper cutting, with the test outcomes serving a secondary role, the inclusion of children aged 9 and 10 years was deemed necessary.

However, these ceiling effects for both duration and mistakes might also reflect the inherent constraints of the task. As Garin (2014) notes, ceiling effects often arise when individuals perform near the upper limit of a test’s capabilities. In this case, children may be executing the task as quickly and accurately as their developmental stage and task demands allow. This interpretation is further supported by the observation that the largest performance gains, both in speed and accuracy, occur between the youngest age groups. This suggests that fundamental skill acquisition occurs relatively early, after which improvements become less pronounced. These findings indicate that task duration, as measured in this study, might be particularly sensitive to delays in younger children but becomes less effective in differentiating performance levels in older children. This could lead to a high number of false negatives in older age groups if task duration is used as the sole indicator of motor skill difficulties. Similarly, while the number of mistakes showed a slightly later ceiling effect, it may also be less effective in detecting subtle improvements or variations in skills beyond a certain developmental stage. In clinical practice, understanding both the quality and quantity of performance is crucial for diagnosing and providing individualised treatment for children with motor difficulties. While older children without motor issues may perform similarly in terms of speed and accuracy, their qualitative strategies might still differ. These differences can offer valuable insights into the underlying motor challenges that are not always apparent in more generalised tests. For example, children who do not demonstrate the most efficient way of moving may tire more quickly, a factor that may not be evident in short tasks but could become relevant in longer and more demanding activities. Recognising such differences can help identify subtle motor issues that could impact daily functioning and guide the development of tailored interventions. This highlights the importance of considering both the quantitative and qualitative aspects of performance. As we have shown, even when quantitative measures plateaued, the qualitative performance of actions within intra-task components continued to change with age. This observation of ceiling effects in a fine motor task like paper cutting aligns with the findings from broader motor skill assessments. For example, French et al. (2018) reported significant ceiling effects in several items of the Movement Assessment Battery for Children-2 (MABC-2; S. E. Henderson et al., 2015), a widely used tool for assessing motor competence in children. Their study highlighted that the presence of ceiling effects can limit the ability of quantitative measures to accurately capture the full spectrum of motor skill development, particularly in typically developing children who perform at higher levels than their peers. Similar to our findings, they emphasised the need for caution when interpreting quantitative data from such assessments. In our previous study on the development of Hands-On! We observed significant moderate correlations between our qualitative observation score and both the duration of the task and the number of mistakes made during the paper-cutting task. These findings suggest that children who perform better in terms of cutting quality, as assessed by Hands-On! tend to spend less time on the task and make fewer errors on the DCDDaily task (Faber et al., 2025). However, it remains unclear whether this relationship holds true for children with developmental disorders, highlighting the need for further research to explore both the quality and quantity of performance in these children. In conclusion, integrating both quantitative and qualitative data offers a more comprehensive understanding of motor skill development.

### 4.3. Strengths, Limitations, and Implications

The current study demonstrates notable strengths that contribute significantly to the understanding of paper cutting among 5- to 10-year-old school-aged children. The use of the newly developed observation tool, Hands-On! in the present study deepens our understanding of age-related changes in the qualitative performance of paper cutting and demonstrates the importance of investigating the quality of fine motor performance. Studies on the qualitative performance of fine motor skills are under-represented in the literature. By focusing specifically on paper cutting, this research highlights its importance in addressing a significant gap in the existing body of knowledge. Furthermore, by comparing our qualitative findings with the quantitative results, we observed a potential ceiling effect in the quantitative data that was not evident in qualitative observations. This suggests that the qualitative measures captured nuances of motor performance that were not detectable by the quantitative measures, highlighting the value of incorporating qualitative analysis into motor skill assessments. Future research should further explore the complementary nature of these methodologies to provide a more comprehensive understanding of motor development.

This study utilised the Hands-On! qualitative observation tool to assess the paper-cutting performance. However, we should acknowledge that more advanced methods for evaluating fine motor skills are available, including writing tablets and joint kinematics using sensors (Clark et al., 2021). While these technologies offer promising insights, they also have certain limitations compared to tools like Hands-On! One major drawback is portability—many of these advanced technologies, mainly the sensor or 3D analysis, are confined to laboratory settings and lack the flexibility needed for widespread clinical or educational use. An exception in this case is writing tablets; however, these writing tablets are only feasible for writing and drawing assessments. Additionally, ensuring sufficient equipment availability and training physicians and therapists to use it effectively may not be practical (Clark et al., 2021). In contrast, the Hands-On! tool integrates seamlessly into daily practice, serving as a structured extension of existing assessments. It is freely accessible, applicable across different observational tasks, and capable of distinguishing between various age groups (Faber et al., 2025). Future research should explore whether this tool can also reliably differentiate between children with motor skill difficulties and typically developing peers.

The current results are cross-sectional and thus capture performance at a single point in time. Despite having a substantial sample size comprising 178 children aged 5- to 10-years-old, a longitudinal approach is essential to fully understand the dynamic and non-linear nature of paper cutting, including how children adapt to task demands and environmental constraints over time. This study sets the stage for future research to track these changes longitudinally, providing a richer and more comprehensive picture of fine motor skill acquisition.

Additionally, the current study utilised the Kruskal–Wallis test to compare the components and actions across different age groups. This approach allowed us to analyse the distributions of observed behaviours while accounting for the transformed categorical data. However, a limitation of this method, particularly given the multiple components that we compared, is the increased risk of Type 1 errors associated with multiple testing, as we did not apply a correction at this stage due to the exploratory nature of this study (Armstrong, 2014). While alternative statistical tests such as Pearson’s chi-square or Fisher’s exact test were considered, they require minimum expected frequencies in all cells, which were not always met in our dataset. Future research with more focused age ranges and larger sample sizes is needed to confirm our initial observations and should consider methods to control for multiple comparisons.

Furthermore, to this point, this study has exclusively focused on typically developing children. However, there is a pressing need to extend the focus of research to children with developmental disorders. Exploring the qualitative performance of paper cutting in children with developmental disorders could demonstrate how their motor skill challenges, as often identified through quantitative tests, manifest in qualitative performance assessments, such as Hands-On!. While quantitative assessments may identify *that* a motor difficulty exists, qualitative analysis reveals how it impacts movement patterns and behaviours. This crucial information is currently lacking in many developmental disorders. Future research should address whether the qualitative fine motor skill performance of children with developmental disorders aligns with or differs from that of typically developing children or even varies between different disorders. Assessing these qualitative profiles can significantly improve our understanding of the unique motor challenges faced by children with various developmental disorders. Additionally, a qualitative approach could help identify unnoticed motor problems that may not be captured by traditional quantitative assessments, providing a more comprehensive understanding of motor development in this population.

## 5. Conclusions

In conclusion, this study provides novel insights into qualitative fine motor performance by examining the cross-sectional development of paper cutting among 5- to 10-year-old typically developing children using the Hands-On! observation tool. Our findings demonstrated age-related differences in actions within intra-task components, often highlighting a progression from less proficient to more proficient actions across age groups. Notably, we identified specific co-occurrences of several actions within the intra-task components, such as the simultaneous use of big cutting movements combined with the coordinated steering of the supporting hand (bi-manual hand use). This contributed to more controlled cutting in older children, highlighting the dynamic interplay between fine motor actions. Furthermore, by comparing the qualitative and quantitative findings, we observed a ceiling effect in the quantitative data that was not evident in the qualitative observations. This suggests that qualitative measures capture the nuances of motor performance that quantitative measures may overlook, reinforcing the importance of integrating both approaches. By focusing on the “how” (qualitative aspects) rather than just the “what” (quantitative aspects) of performance, this study lays the groundwork for future research comparing typical and atypical (motor) development, ultimately informing diagnostic processes and targeted interventions.

## Figures and Tables

**Table 1:** Descriptive statistics.

	Total Group	5-Year-Olds	6-Year-Olds	7-Year-Olds	8-Year-Olds	9-Year-Olds	10-Year-Olds
	*n* = 178	*n* = 21	*n* = 31	*n* = 37	*n* = 32	*n* = 34	*n* = 23
Age (years) ^1^	8.06 ± 1.58	5.53 ± 0.34	6.55 ± 0.27	7.47 ± 0.25	8.59 ± 0.25	9.52 ± 0.30	10.44 ± 0.30
Sex (% boys) ^2^	47.8 (*n* = 85)	38.1 (*n* = 8)	51.6 (*n* = 16)	45.9 (*n* = 17)	50.0 (*n* = 16)	61.8 (*n* = 21)	30.4 (*n* = 7)
Handedness (% right-handed) ^2^	87.6 (*n* = 156)	85.7 (*n* = 18)	90.3 (*n* = 28)	91.9 (*n* = 34)	81.3 (*n* = 26)	82.4 (*n* = 28)	95.7 (*n* = 22)
Cutting time (s) ^3^	73.0 (57.0, 91.3)	90.0 (77.0, 123.0)	74.0 (57.0, 89.0)	74.0 (57.5, 95.5)	73.0 (57.3, 88.8)	66.0 (55.0, 88.3)	65.0 (53.0, 75.0)
N mistakes ^3^	1.0 (0.0, 3.0)	6.0 (2.50, 7.5)	3.00 (1.0, 4.0)	1.0 (0.0, 4.0)	1.0 (0.0, 1.0)	0.0 (0.0, 2.0)	0.0 (0.0, 1.0)

*Note.* ^1^ Mean and standard deviation, ^2^ Percentage and frequency, ^3^ Median and interquartile range.

**Table 2:** Intra-task components and actions for the paper-cutting task.

Intra-Task Component	Actions
Manipulation	No manipulation In-hand manipulation Transferring hand-to-hand Hand, body or surface assist.
Grip type scissors	Thumb above, middle finger lower hole, index finger supports bottom scissors Thumb above, middle and index finger lower hole Thumb above, index finger lower hole Thumb lower hole, middle and or index finger above
Grip type fingers	Fingers fold around scissors Fingers fold around scissors, thumb is extended Fingers stick out the side
Cutting movements	Large cutting movements Small cutting movements
Closing the scissors	Does not fully closes the scissors Fully closes the scissors
Fluency	Fluent Not fluent
Arm position	Arms are in slight abduction, no table support Arms are in slight abduction, table support Arms are pressed against the body, no table support Arms are pressed against the body, table support Arms are in abduction, no table support Arms are in abduction, table support
Supporting hand	Active in the air Passive/stiff in the air On the table
Posture	Sits upright or is slightly bend over Is bend over a lot Hangs or leans back

*Note.* For a complete description of the components and actions, as well as picture and video examples, see the Supplementary Material for the link to Hands-On!.

**Table 3:** Significant differences in the percentage occurrence per component and actions across age groups.

Components	Action	H(5)	*p*-Value	η^2^
Grip type scissors	Thumb in the top hole, middle finger in the bottom hole, index finger supports	11.576	0.041	0.027
Grip type fingers	Fingers fold around the scissors	25.105	<0.001	0.105
	Fingers fold around the scissors but thumb is extended	19.094	0.002	0.070
	Fingers stick out the side	11.108	0.049	0.024
Cutting movement	Big cutting movements	19.762	0.001	0.074
	Small cutting movements	19.762	0.001	0.074
Closing the scissors	Closes the scissors completely	27.842	<0.001	0.121
	Does not completely close the scissors	27.842	<0.001	0.121
Fluency	Fluent	19.291	0.002	0.071
	Not fluent	19.291	0.002	0.071
Arm position working hand	Slight abduction, no table support	23.190	<0.001	0.094
	Arm in abduction not on the table	20.004	0.001	0.076
Arm position supporting hand	Slight abduction, no table support	13.229	0.021	0.036
	Pressed against the body, supported on the table	15.037	0.010	0.047
Supporting hand	Active in the air	13.764	0.017	0.039
	Passive or stiff in the air	13.695	0.018	0.039

*Note.* H = Kruskal–Wallis test; df = 1. Effect size η^2^ defined by: η^2^ < 0.059 indicates a small effect; η^2^ between 0.060 and 0.139 indicates a moderate effect, and η^2^ > 0.140 indicates a large effect.

## Data Availability

The data presented in this study are available upon request from the corresponding author. The data are not publicly available because parental consent was only given for the use of such data by the researchers directly involved in the ‘Uniek in je Motoriek’ [Being unique with regard to your motor skills] project.

## Supplementary Materials

The Hands-On! qualitative observation tool for fine motor skills can be downloaded at: https://zenodo.org/records/14185207 (accessed on 1 January 2025).
